# Pregnancy in a Unicornuate Uterus with Non-Communicating Rudimentary Horn: Diagnostic and Therapeutic Challenges

**DOI:** 10.15388/Amed.2020.27.2.6

**Published:** 2020-12-21

**Authors:** Ratko Delić

**Affiliations:** Department of Obstetrics and Gynecology, General and Teaching Hospital Celje, Slovenia

**Keywords:** infertility, unicornuate uterus, pregnancy, cervical incompetence, sepsis

## Abstract

Unicornuate uterus with non-communicating rudimentary horn is a type of congenital uterine abnormality that occurs as a consequence of the arrested development of one of the two Müllerian ducts.

Patients with unicornuate uterus have increased incidence of obstetric and gynaecological complications.

We present a report of a clinical case of a 28-years-old female, who was referred to the hospital for evaluation of her infertility.

The patient reported primary infertility and inability to conceive after 3-year period of regular unprotected intercourse.

Transvaginal ultrasound, along with the preoperative evaluation were completed; however, no anomalies or irregularities were reported.

Combined diagnostic simultaneous laparoscopy and hysteroscopy were performed to establish the diagnosis of unicornuate uterus with non-communicating rudimentary horn.

The patient conceived spontaneously after diagnostic laparoscopy and hysteroscopy.

During and after pregnancy, our patient and her child experienced numerous complications (cervical incompetence, acute chorioamnionitis, acute fetal distress, pneumonia, septic shock) and procedures (cervical cerclage, urgent cesarean section, intensive care unit treatment) without significant fetal or maternal compromise.

## Abstract (lt)

**Nėštumas vienaragėje gimdoje su rudimentiniu nesusijungusiu ragu:**

Vienaragė gimda su rudimentiniu nesusijungusiu ragu yra įgimta makšties anomalija, atsiradusi sutrikus vieno iš dviejų Miulerio latakų vystymuisi.

Pacientės, kurių gimda yra vienaragė, dažniau patiria gimdymo ir ginekologinių komplikacijų.

Pristatome klinikinio atvejo – 28 metų amžiaus moters, kuri buvo siunčiama į ligoninę dėl nevaisingumo, ataskaitą.

Pacientė pranešė apie pirminį nevaisingumą ir negalėjimą pastoti po 3 metų reguliarių lytinių santykių be apsaugos priemonių.

Atliktas transvaginalinis ultragarso tyrimas ir priešoperacinis įvertinimas, tačiau nenustatyta jokių anomalijų ar nukrypimų nuo normos.

Atlikta diagnostinė laparoskopija ir histeroskopija: diagnozuota vienaragė gimda su rudimentiniu nesusijungusiu ragu.

Pacientė, atlikus diagnostinę laparoskopiją ir histeroskopiją, pastojo pati.

Nėštumo metu ir po jo pacientė ir jos vaikas patyrė daugybę komplikacijų (gimdos kaklelio nepakankamumas, ūmus chorioamnionitas, ūmi vaisiaus hipoksija, plaučių uždegimas, sepsinis šokas) ir jiems buvo atliktos įvairios procedūros (gimdos kaklelio apsiuvimas, skubi cezario pjūvio operacija, gydymas intensyvios priežiūros skyriuje), nepakenkiant nei vaisiaus, nei motinos sveikatai.

**Raktažodžiai:** nevaisingumas, vienaragė gimda, nėštumas, gimdos kaklelio nepakankamumas, sepsis

## Introduction

Unicornuate uterus with non-communicating rudimentary horn is a Müllerian anomaly, which results from the normal differentiation of only one Müllerian duct.

Women with unicornuate uterus have increased incidence of obstetric complications (recurrent miscarriages, ectopic pregnancy, prematurity, intrauterine growth restriction, antepartum & postpartum bleeding, cervical incompetence, malpresentation, pregnancy-associated hypertension, operative delivery, uterine rupture, intrauterine fetal demise) and gynaecological complications (infertility, dysmenorrhoea, chronic pelvic pain) [1].

Here, we present a case of pregnancy in a unicornuate uterus with non-communicating rudimentary horn and its peculiarities.

## Case presentation

A 28 years-old nulligravida was referred to our department for laparoscopic and hysteroscopic evaluation of her infertility.

Diagnostic laparoscopy and tubal patency test revealed unicornuate uterus with non-communicating rudimentary horn on the right side, confirming left tubal patency. Macroscopically, both ovaries were of normal appearance. (ESHRE/ESGE classification system for female genital malformations Class IV b or Class II C by the American Fertility Society) [2, 3].

**Figure 1.**
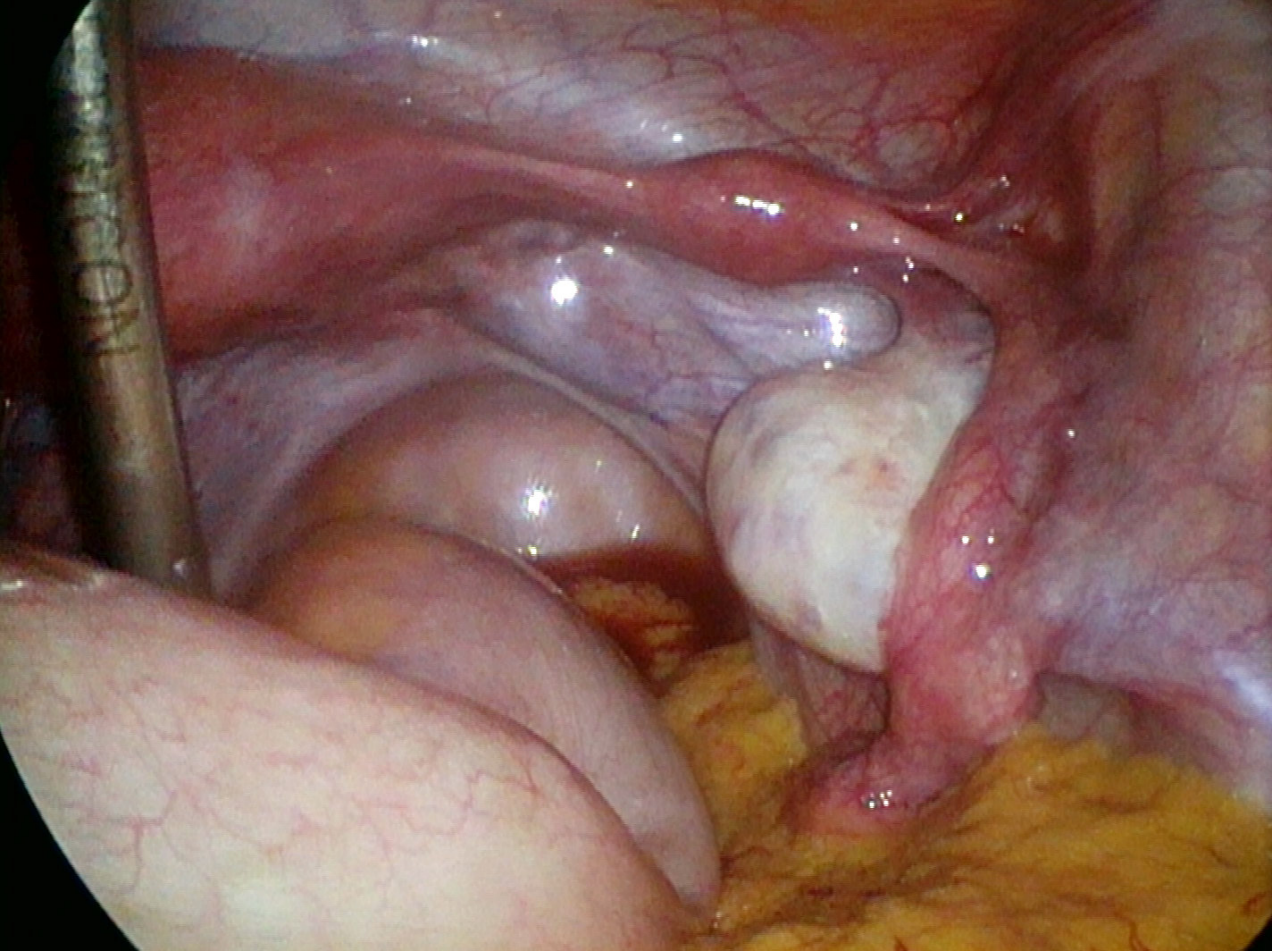
Laparoscopy image of the unicornuate uterus with a non-communicating right rudimentary horn.

Visual inspection with hysteroscopy demonstrated only left tubal ostium.

She became spontaneously pregnant several months after diagnostic laparoscopy and hysteros-copy.

Nonetheless, 21 weeks into the pregnancy, the patient was once again referred to our department with a diagnosis of cervical incompetence (a cervix less than 20 mm).

The McDonald cervical cerclage was performed and tocolytic therapy was prescribed.

The cerclage was removed electively at 37 weeks of gestation.

At 40 weeks pregnant, the patient came to our department complaining of contractions, rupture of the membranes, shortness of breath, coughing and fever (> 38°C).

Vaginal examination revealed foul smelling meconium-stained amniotic fluid while CTG recordings were pathological.

The patient underwent urgent caesarean delivery; a male infant was born with an Apgar score of 7, 5 and 9, weight of 2800g and height of 53 cm.

Prior to intensive care unit (ICU) admission, the patient was treated with broad-spectrum antibiotics and antihypertensive drugs resulting in clinical deterioration (worsening of the hypoxaemia despite oxygen therapy, high inflammatory markers - leukocytes 13,0 10^9/L, C-reactive protein 267,7 mg/L, low haemoglobin level 84 g/L).

The patient’s chest X-ray demonstrated bilateral pleural effusion as well as consolidation in both lobes.

After three days of respiratory support, pleural aspiration (1200 ml), red blood cell transfusion, antihypertensive therapy and treatment with azithromycin 500 mg once daily and piperacillin/tazo-bactam 4,5 grams three times daily the patient’s clinical condition improved.

The infant was discharged after a twelve days course of intravenous antibiotics, and follow-up examinations over a three-year period revealed a healthy and neurologically intact child.

**Figure 2.**
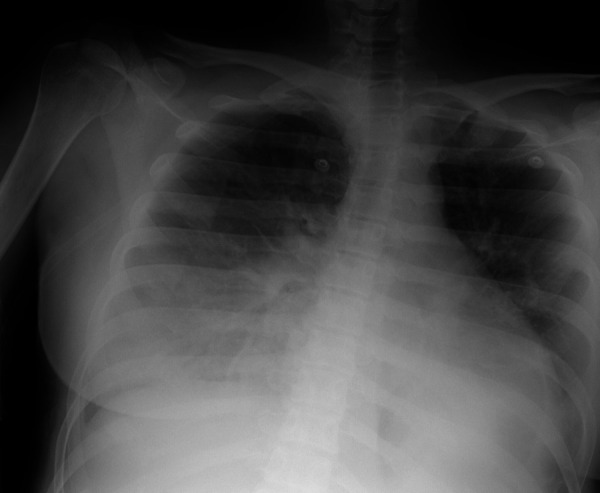
Chest X-ray of the patient showing bilateral lower lobe consolidation with pleural effusion.

## Discussion

An exact prevalence of uterine anomalies is difficult to establish since many are never diagnosed, especially if they are asymptomatic. Reported prevalence varies depending on the population studied. [4, 5, 6].

According to Chan et al., the prevalence of uterine malformations is estimated to be 5.5% in general population and at approximately 8% in women with infertility (4).

Unicornuate uterus is relatively uncommon, representing 2.5–13.2% of all uterine malformations [4].

The conventional methods for the assessment of Müllerian anomalies are two-dimensional (2D) ultrasound, hysterosalpingography, hysteroscopy and laparoscopy.

With the fast improvement of volume ultrasound, three-dimensional (3D) ultrasound has been accepted as additional gold standard for the diagnosis of Müllerian anomalies, nonetheless, ultrasound diagnosis can be easily missed, as in our case [7].

Magnetic resonance imaging should be considered an addition to ultrasound to assess Müllerian anomalies [8].

A diagnosis of unicornuate uterus is often demanding and delayed to the fertile period.

The unicornuate uterus may occur alone, but it is usually associated with a rudimentary horn [9].

The majority of rudimentary horns are asymptomatic, as in our case, but others contain functional endometrium that can nurture an ectopic pregnancy or develop cyclic or chronic pelvic pain if the rudimentary horn is obstructed [10].

However, surgical correction of a unicornuate uterine anomaly is not warranted in asymptomatic women or those with primary infertility therefore we decided not to perform the resection of the rudimentary horn [10].

Several studies of uterine anomalies and pregnancy outcomes revealed that the unicornuate uterus had the poorest overall reproductive outcomes of all the uterine anomalies [9, 10].

Analysis of nearly 400 pregnancies revealed the following outcomes for the entire unicornuate class: 43.3% preterm deliveries, 54.2% live births, 4.3% ectopic pregnancies, and 34.4% miscarriages [11].

Reichmann and colleagues in their review reported the following rates of pregnancy outcomes in unicornuate uteri: 2.7% ectopic pregnancy, 24.3% first trimester miscarriage, 9.7% second trimester miscarriage, 20.1% preterm delivery, 3.8% intrauterine fetal demise, and 51.5% live births [12].

Problems with reproduction were attributed to abnormal uterine vasculature and reduced myo-metrial mass of the unicornuate uterus [13].

The presence of only one uterine artery and the limited contribution of the contralateral arterioles compromises the blood supply, diminishing the muscular mass of the unicornuate uterus.

This diminished muscle mass is thought to play an important role in the occurrence of isthmus-cervical incompetence.

Airoldi and colleagues reported that unicornuate uterus had the highest rate of cervical shortening and preterm delivery of all uterine anomalies [14].

Cervical cerclage has been placed in many patients with unicornuate uterus often successfully; however, routine placement of cervical cerclage early in pregnancy is controversial [12].

It is important to consider optimal mode of delivery based on the findings and the anticipated duration until birth, however, expedite delivery is warranted if there are signs of acute chorioamnio-nitis, as in our case.

Approximately 20–30% of intensive care unit admissions of obstetric patients result from sepsis in pregnancy [15].

Fortunately, after treating underlying infection with a potent antibiotic combination and supporting failing organ functions, mother and child made a full recovery from childbirth.

In conclusion, despite the fact that 3D transvaginal ultrasound has become nowadays the method of choice in assessing the uterus due to many advantages over all other techniques, ultrasound diagnosis can be easily missed, especially in inexperienced hands [5].

This case is distinctive because the patient and her child experienced numerous difficulties, procedures and complications before (infertility, diagnostic laparoscopy and hysteroscopy), during (cervical incompetence, acute chorioamnionitis, pneumonia, septic shock, cervical cerclage, urgent cesarean section) and after pregnancy (ICU supportive care).

It demonstrates why women with a unicornuate uterus with non-communicating rudimentary horn should be considered as high-risk patients.
